# Digit ratio (2D:4D), laryngeal cancer and vocal fold leukoplakia

**DOI:** 10.1007/s00432-023-04850-8

**Published:** 2023-05-21

**Authors:** Wioletta Pietruszewska, Joanna Morawska, John T. Manning, Aneta Sitek, Bogusław Antoszewski, Anna Kasielska-Trojan

**Affiliations:** 1 Department of Otolaryngology, Head and Neck Oncology, Medical University of Lodz, Lodz, Poland; 2 Applied Sports, Technology, Exercise, and Medicine (A-STEM), Swansea University, Swansea, UK; 3 Department of Anthropology, University of Lodz, Lodz, Poland; 4 Plastic, Reconstructive and Aesthetic Surgery Clinic, Institute of Surgery, Medical University of Lodz, Kopcinskiego 22, 90-153 Lodz, Poland

**Keywords:** Laryngeal cancer, Leukoplakia, Digit ratio, Risk factors

## Abstract

**Background:**

To date, there are no studies that have analyzed the possible influence of exposure to prenatal sex hormones on the risk of laryngeal cancer (LC) and premalignant laryngeal lesion—vocal fold leukoplakia (VFL). Digit ratio (2D:4D) is suggested to be a proxy of prenatal sex hormone exposure.

**Objective:**

To examine 2D:4D in patients with LC and clarify if it could add to the verified risk factors in estimating the overall risk of LC.

**Methods:**

511 subjects participated in the study. The study group included 269 patients: with LC (*N* = 114, 64 men) and VFL (*N* = 155, 116 men). Controls included 242 healthy individuals (66.40 ± 4.50 years (106 men)).

**Results:**

Predictive models estimating the risk of VFL and LC in women, based solely on predictors like smoking and alcohol consumption had a lower area under the ROC curve (AUC) than the model with left 2D:4D. AUC for the model estimating the likelihood of VFL increased from 0.83 to 0.85, and for LC from 0.76 to 0.79.

**Conclusions:**

Low left 2D:4D may be associated with an increased risk of developing leukoplakia and laryngeal cancer in women. In the case of laryngeal cancer, left 2D:4D may serve as additional variable (to other known risk factors, such as smoking and/or alcohol consumption), which can improve cancer risk prediction.

## Introduction

Cancer is a predominant cause of death and a critical barrier to increasing life expectancy in every country of the world (Bray et al. 2018; Sung et al. 2021). Laryngeal carcinoma (LC) is the eleventh most common form of cancer worldwide and is the second most common malignancy of the head and neck (Chu and Kim 2008) with five-year relative survival estimated at 61% (Gatta et al. 2017)**.** An increase in the incidence of LC has been noted over the last five decades, mainly among the male population (Johnson et al. 2020), despite the development of new diagnostic and therapeutic techniques.

It is estimated that about 90% of malignant tumors of the larynx arise from premalignant laryngeal lesions (PLL). These are identifiable local lesions which tend to transform into invasive carcinoma (Avila et al. 2014). Vocal fold leukoplakia (VFL) is the most common precancerous lesion in otolaryngological practice. It presents as thick whitish or gray patches on the epithelium and represents a variety of lesions without considering their etiology and histopathological features (Huang et al. 2017; Li et al. 2018; Singh et al. 2014; Wan et al. 2021).

The etiology of LC and PLL is multifactorial, with both genetic and environmental factors participating in the development of the disease. The main oncogenic factors include long-term tobacco use, alcohol consumption and infection with high-risk types of Human Papilloma Virus (HPV) (Alsahafi et al. 2019). A significantly higher male to female ratio in LC and PLLs suggests the involvement of gender-dependent factors in the pathogenesis. There is epidemiologic, clinical, and experimental evidence suggesting that sex hormones play an important role in development and progression of some cancers: breast, prostate and testicular (Bunevicius 2018). It is also considered that events and environment (including hormones) during prenatal period, when organs are developing, can increase cancer risk in adult life (Grotmol et al. 2006).

To date, there are no studies that have analyzed the possible influence of exposure to prenatal sex hormones on the risk of laryngeal cancer (and precancerous lesions such as leukoplakia) occurrence. However, such direct study would be impossible, due to ethical reasons concerning the detrimental influence of very early fetal examination, and the fact that not all humans who later develop cancer have been screened in utero. That is why we can look only in retrospect at a biomarker that correlates with prenatal sex steroids, so we used a putative proxy of prenatal sex hormone exposure. The ratio of the lengths of the second digit and fourth digit (digit ratio or 2D:4D) is sexually dimorphic (2D:4D males < 2D:4D females). It has been reported that 2D:4D is a biomarker of prenatal sex steroids exposure—low ratio correlates with high prenatal testosterone and low estrogens, while high ratio is the effect of low fetal testosterone and high estrogens (Manning et al. 1998; Manning 2011; Manning and Fink 2018). Also, right-left 2D:4D (Dr–l) has been suggested to be a negative correlate of high prenatal testosterone and low prenatal estrogen (Manning 2002, 2008). Evidence supporting the relationships between 2D:4D and prenatal sex steroids in humans has been recently reviewed by Swift-Gallant et al. (2020) (Swift-Gallant, Johnson, Di Rita, and Breedlove, 2020) and evidence against by McCormick and Carré (2020) (McCormick and Carré, 2020). Experimental evidence regarding links between 2D:4D and prenatal sex hormones has been reported by Zheng and Cohn (2011) in mice (Zheng and Cohn 2011) and by Auger et al. (2013) in rats (Auger et al. 2013). They found that sex differences in 2D:4D are determined by a balance of prenatal testosterone to estrogen in a narrow time window of fetal digit development.

There are some studies suggesting that 2D:4D ratio can serve as a biomarker for sex hormones-dependent cancers. Prenatal exposure to sex steroids may modulate the risk of disease occurrence in adulthood, which may be related to the cumulative effect of carcinogens and hormonal stimulation. A meta-analysis revealed that the sex hormone environment during early development may be associated with cancer risk later in life as low 2D:4D was associated with: prostate cancer, gastric cancer, and brain tumors risk, while high 2D:4D, was linked to breast cancer and cervical dysplasia (Bunevicius 2018; Hong et al. 2014). Recent studies showed that low (“masculinized”) right 2D:4D and Dr–l are associated with a predisposition to lung cancer and/or the more aggressive forms of lung cancer in women. In the light of these findings, the authors hypothesize that women with lower prenatal estrogens (low 2D:4D) are more prone to cancer due to developmental inhibition of the respiratory system. This results in more “vulnerable” lungs and/or less protective function of estrogens on lung epithelium (Kasielska-Trojan et al. 2020). A report of a negative association between 2D:4D and risk of developing oral cancer in women may be further evidence for this association (Ying et al. 2022). The role of 2D:4D as a marker of prenatal sex hormones, the sex hormone-dependent character of laryngeal cancer and correlations presented in the literature for oral and lung cancer (which are also connected with the influence of external carcinogens such as smoking and alcohol consumption), encouraged a hypothesis that: (1) women and men with laryngeal cancer may present lower (more masculinized) digit ratio, (2) the influence of prenatal sex hormone exposure could add to the verified risk factors of laryngeal cancer (smoking, drinking alcohol, HPV infection) in estimating overall risk of laryngeal cancer, (3) similar effects would be observed in patients with premalignant laryngeal lesions such as leukoplakia.

## Materials and methods

### Participants

A total of 511 subjects participated in the study. The study group included 269 patients, 89 women and 180 men, aged from 20 to 82 (mean age 54.7) diagnosed with laryngeal cancer (LC) (*N* = 114, 64 men, 50 women) and vocal fold leukoplakia (VFL) (*N* = 155, 116 men, 39 women). The patients were examined at the Otolaryngology, Head and Neck Oncology Department of the Medical University and in University Hospital Outpatient Clinic in the period from 2017 to 2021.The inclusion criteria were diagnosed LC or VFL, no prior vocal fold-related medical intervention, and procedures (surgery, radiation), and pre-operative endoscopic assessment by means of videolaryngoendoscopy (VLE); no hand injuries in anamnesis, without congenital abnormalities and hormonal disturbances. The exclusion criteria were: the disqualification of the patient from direct laryngoscopy with the collection of material for histopathological examination, e.g., when conservative treatment was implemented, patients who had previously undergone surgical interventions in the vocal folds or were diagnosed with leukoplakia in a location other than the glottis.

In the group of patients with LC there were 33 (32.7%) subjects with T1/T2 and 68 (67.3%) subjects with T3/T4. Nodal involvement (N1, N2, N3) was observed in 36 (35.6%) patients. TNM classification for the examined group of LC patients revealed stage I in 15 (14.8%) patients, stage II in 18 (17.8%) patients, stage III in 24 (23.8%) patients and stage IV in 44 (43.6%) patients. Regarding the histological type of tumor squamous cell carcinoma was confirmed in all cases with G1 in 25 (24.8%), G2 in 56 (55.4%) and G3 in 20 (19.8%) cases. In the patients with vocal fold leukoplakia 54 (65%) cases were classifies as low-grade dysplasia and 29 (35%) as high-grade dysplasia in histopathological examination.

The control group comprised 242 healthy individuals, normophonic subjects aged 66.40 ± 4.50 [mean ± SD] years, 136 women and 106 men recruited from Plastic Surgery Clinic patients. The inclusion criteria were: no laryngological symptoms, no vocal com-plaints, no history of laryngeal disorders, no hand injuries in anamnesis, without congenital abnormalities and hormonal disturbances.

The protocol of the study included a questionnaire (sex, age, age at diagnosis, stage of the disease, histopathological diagnosis, history of smoking (all active and past smokers with at least cumulative pack-years over 10 were included as “smokers”) and alcohol consumption (referred to current practices: no/never and occasional as “No” and more than 3 times/week, regularly as “Yes”), and anthropometric measurements. All participants of the study completed a questionnaire and anthropometric measurements were performed directly rather than from indirect images from photocopies or scans. All the participants were Polish (inclusion criterion, based on medical charts).

Approval for this study was granted by the Ethical Committee of the Medical University of Lodz (decision no RNN-96/20KE) and informed consent was obtained from each patient before inclusion. Confidentiality was ensured by a numerical cross-referencing system.

## Measurements

The following anthropometric measurements were obtained for all the participants: second- and fourth-digits’ lengths (2D and 4D) (right (R) and left hand (L)). Each measurement was taken twice by the same individual, and the mean of the two measurements was considered as the final reading. Based on these parameters, the following variables were calculated: 2D:4D for the right (R) and left (L) hand (2D length [mm]/4D length [mm]) (2D:4D R, 2D:4D L) and right minus left 2D:4D (Dr–l). All measurements were made directly with sliding Vernier-type calliper (*GPM anthropometric instruments*) with accuracy of 0.1 mm. Measurements were performed on the palmar side of the hand using anthropometric points on the digit axis: pseudophalangion–a point in the finger metacarpophalangeal crease, dactylion–the most distal point on the fingertip (Kasielska-Trojan and Antoszewski 2015).

### Statistical analysis

To compare quantitative variables (age at the time of examination and diagnosis, digits lengths, 2D:4D ratios and Dr–l) between participants with laryngeal cancer, leukoplakia and the control group, one-way analysis of variance (ANOVA) or its non-parametric equivalent—Kruskal–Wallis test (depending on the normality of the distribution and homogeneity of the variance) was used. Effect sizes were calculated for each comparison: omega-square for one-way ANOVA or epsilon-squared for Kruskal–Wallis test. If the intergroup comparisons were statistically significant, the post hoc tests were used (Tukey test for unequal N for one-way ANOVA or multiple comparisons of mean ranks for the Kruskal–Wallis test). Correlations between age at the time of the study and age at diagnosis and right and left 2D:4D and Dr–l were examined using the Spearman's rho coefficient (due to the lack of normality of distribution of both analyzed ages). Sexual dimorphism of 2D: 4D R and L and Dr–l was assessed with t-tests for equal or different variances. The frequency of using smoking and alcohol consumption in the analyzed groups was compared using the fraction test, with Bonferroni correction (for multiple comparisons). The relationships between R and L 2D: 4D and Dr–l and the risk of laryngeal cancer and leukoplakia were analyzed using logistic regression models, each time with adjustment for smoking and alcohol consumption. The quality of each logistic regression model was characterized by the R^2^ Nagelkerke’s coefficient. When 2D:4D or Dr–l turned out to be a significant predictor of laryngeal cancer or leukoplakia, the area under the ROC curve was compared for the model containing this predictor adjusted for smoking and alcohol consumption with the area under the ROC curve for the model based only on smoking and alcohol consumption (hierarchical models). Comparisons of AUC for both models were made based on J. Haney's algorithm implementing the method described by (Hanley and Hajian-Tilaki 1997). The ROC (Receiver Operating Characteristic) curve was used to evaluate the model. It presents the relationship between the sensitivity and specificity for the probability of the event estimated by the model. The area under the ROC curve—AUC (area under curve)—is determined as a measure of accuracy of the given model. The values of AUC range from 0 to 1 and the larger the AUC, the better the model. There are no universal guidelines as to how to interpret the individual AUC values, but the following classification is often applied: AUC < 0.5 false classifier (worse than random), 0.5–random classifier, 0.5 < AUC < 0.7—insufficient discrimination, 0.7 ≤ AUC ≤ 0.8–acceptable discrimination, AUC > 0.8–perfect discrimination.

Statistical analysis was performed using IBM SPSS Statistics version 13.3.

### Power analysis

For the power calculations, we applied an elementary approach with power calculation for the two-sample t-test, using the estimated difference in mean (delta) and standard deviations (SD). We set the standard significance level for 0.05. For the posteriori analysis of power, we used a Gaussian prior for the delta and an inverse Gaussian prior for the SD (k = 1000 repetitions). We used data from three studies chosen from Fonseca et al (2022): Wang et al. (2018) (delta 1 = 0.98–0.96 = 0.02, SD 1 = 0.04, Bandwidth = 0.03775; delta 2 = 0.98–0.95 = 0.03, SD 2 = 0.037, Bandwidth = 0.03216), Hopp et al. (2015) (delta 1 = 0.97–0.95 = 0.02, SD 1 = 0.044, Bandwidth = 0.07807; delta 2 = 0.97–0.95 = 0.02, SD 2 = 0.041, Bandwidth = 0.07232) and Kasielska‑Trojan et al. (2020) (delta 1 = 0.997–0.973 = 0.024, SD 1 = 0.037, Bandwidth = 0.0642; delta 2 = 0.97–0.978 = -0.008, SD 2 = 0.04, Bandwidth = 0.07862). As far as the sample size in the current study is concerned, in all the above cases, the posterior power is greater than 0.8 for *n* = 250.

## Results

Table 1 summarizes the means and standard deviations of all analyzed variables (age at the time of the study, age at diagnosis, the length of R and L 2D and 4D, R and L 2D:4D and Dr–l). There were no significant differences in the age during the study between the control group and patients with laryngeal cancer and leukoplakia, both for men and women. However, significant differences were noted for the age at diagnosis—men with cancer and leukoplakia at the time of diagnosis were significantly younger than control men at the time of the study. Among women, only patients with leukoplakia at the time of diagnosis had a lower average age than the control group. For both sexes, no age differences at the time of diagnosis were found between the groups (Table 2). The mean age of men and women at the time of diagnosis (laryngeal cancer or leukoplakia) and 2D:4D and Dr–l did not correlate (Table 3).

**Table 1:** Mean age, digit measurements, 2D:4D ratios and Dr–l of study males and females

Variables	Men *N* = 286						Women *N* = 225					
	Control group *N* = 106		Leukoplakia *N* = 116		Laryngeal cancer *N* = 64		Control group *N* = 136		Leukoplakia *N* = 39		Laryngeal cancer *N* = 50	
	${\overline{\text{x}}}$	SD	${\overline{\text{x}}}$	SD	${\overline{\text{x}}}$	SD	${\overline{\text{x}}}$	SD	${\overline{\text{x}}}$	SD	${\overline{\text{x}}}$	SD
Age [yrs.]^1^	66.05	4.35	64.74	11.08	67.12	10.34	66.40	4.50	64.67	10.32	69.31	11.42
Age [yrs.]^2^	–	–	61.33	10.79	62.38	9.77	–	–	61.38	10.61	64.72	10.96
2DR [mm]	75.8	4.5	75.2	3.9	76.1	3.1	67.3	4.1	66.3	2.1	67.7	4.1
4DR [mm]	78.0	4.6	77.2	3.7	78.2	2.8	68.1	5.0	67.7	2.3	69.1	4.3
2DL [mm]	77.5	4.4	78.2	4.3	76.8	3.9	69.6	4.4	68.6	2.1	69.2	4.3
4DL [mm]	79.2	4.7	80.0	4.1	78.7	3.8	69.7	4.7	69.5	2.6	70.0	4.3
2D:4DR	0.9723	0.032	0.9745	0.021	0.9730	0.02	0.9887	0.037	0.9803	0.024	0.9789	0.03
2D:4DL	0.9794	0.033	0.9773	0.014	0.9758	0.014	0.9991	0.033	0.9861	0.022	0.9889	0.017
Dr–l	−0.0070	0.031	−0.0029	0.012	−0.0028	0.01	−0.0104	0.032	-0.0072	0.03	−0.0086	0.013

${\overline{\text{x}}}$ mean, *SD* standard deviation, *2D* second finger length, *4D* fourth finger length, *2D:4D* second to fourth digit ratio, *R* right hand, *L* left hand, *Dr–l* = *2D:4D R -* 2D:4D L

^1^age at the time of the study

^2^age at diagnosis

**Table 2:** Comparison of age, digit measurements, 2D:4D ratios and Dr–l between patients with leukoplakia or laryngeal cancer and control group

Variables	Men			Women		
	F/H	ω^2^/E^2^_R_	*p*	F/H	ω^2^/E^2^_R_	*p*
Age [yrs.]^1^	0.79*	0.00*	0.6731	5.00*	0.02*	0.0820
Age [yrs.]^2^	12.05*	0.04*	0.0024	14.13*	0.06*	< 0.001
Leukoplakia vs. control group Laryngeal cancer vs. control group Leukoplakia vs. laryngeal cancer			0.0135 0.0071 1.0000			< 0.001 0.2774 0.2104
2DR	1.31	0.00	0.2702	1.58	0.01	0.2081
4DR	2.09	0.01	0.1257	1.10	0.00	0.3332
2DL	2.28	0.01	0.1044	1.07	0.00	0.3447
4DL	2.02	0.01	0.1347	0.13	0.00	0.8813
2D:4DR	0.20	0.00	0.8181	1.98	0.01	0.1401
2D:4DL Leukoplakia vs. control group Laryngeal cancer vs. control group Leukoplakia vs. laryngeal cancer	0.51	0.00	0.6020	4.44	0.03	< 0.05 < 0.05 < 0.05 0.9007
Dr–l	1.29	0.00	0.2773	0.22	0.00	0.8008

^1^age at the time of the study

^2^age at diagnosis

*F* one-way ANOVA, H* Kruskal–Wallis test, *ω*^2^ effect size for ANOVA, *E*^*2*^_R_* effect size for Kruskal–Wallis test, *p* probability

**Table 3:** Correlation between the age at diagnosis and 2D:4DR, 2D:4DL and Dr–l

Sex	age at diagnosis & 2D:4DR		age at diagnosis & 2D:4DL		age at diagnosis & Dr–l	
	*R*	*p*	*R*	*p*	*R*	*p*
Men *n* = 180	-0.009	0.8757	-0.008	0.8924	0.000	0.9935
Women *n* = 89	0.071	0.2907	0.086	0.1978	-0.014	0.8363

*R* Spearman's rank correlation coefficient, *p* probability

When assessing sexual dimorphism of 2D:4D and Dr–l, it was found that right 2D:4D was higher in women compared to men only in the control group. Left 2D:4D turned out to be dimorphic in all analyzed groups, and the direction of these differences was consistent with theoretical expectations (i.e. 2D:4D lower in men than in women). The Dr–l showed sex differences only among patients with laryngeal cancer and turned out to be higher in men than in women (Table 4).

**Table 4:** Sexual dimorphism of 2D:4DR, 2D:4DL and Dr–l

Variables	Men vs. Women					
	Control group		Leukoplakia		Laryngeal cancer	
	*t*	*p*	*t*	*p*	t	*p*
2D:4DR	−3.62	< 0.001	−0.87	0.3897	−1.80	0.0743
2D:4D L	−4.64	< 0.0001	−2.35	< 0.05	−4.43	< 0.0001
Dr–l	0.82	0.4114	0.86	0.3923	2.64	< 0.01

*t* t-test (optional for equal or different variances), *p* probability

Table 5 summarizes the qualitative risk factors for the development of leukoplakia and laryngeal cancer. The fraction test showed that both nicotine and alcohol consumption were more common among patients than in the control group. After applying the Bonferroni correction, the statistical significance was maintained by the differences in smoking; while in the case of alcohol consumption, only women with laryngeal cancer declared it more often than women from the control group. There were no differences in terms of smoking and the frequency of alcohol consumption between participants with leukoplakia and laryngeal cancer (Table 5).

**Table 5:** Analysis of smoking and alcohol consumption

Sex		Groups			Fraction test		
		Control group	Leukoplakia	Laryngeal cancer	Control group vs leukoplakia *p*	Control group vs laryngeal cancer *p*	Leukoplakia vs laryngeal cancer *p*
Men	Total *N* (%)	106 (100%)	116 (100%)	64 (100%)	–	–	–
	Smokers	27 (25%)	90 (78%)	52 (81%)	< 0.0001*	< 0.0001*	0.6358
	Drinking alcohol	44 (42%)	66 (57%)	38 (59%)	< 0.05	< 0.05	0.7949
Women	Total *N* (%)	136 (100%)	39 (100%)	50 (100%)	–	−	–
	Smokers	22 (16%)	30 (77%)	33 (66%)	< 0.0001*	< 0.0001*	0.2574
	Drinking alcohol	39 (29%)	18 (46%)	25 (50%)	< 0.05	< 0.01*	0.7079

*Significant after Bonferroni correction (*p* < 0.025)

### Prediction models including smoking and alcohol consumption and 2D:4D

Multivariate regression showed that in the male group, after adjustment for smoking and alcohol consumption, none of the digit ratios were a significant risk factor for the development of leukoplakia and/or laryngeal cancer (Tables 6, 7). However, in women low left 2D:4D (with smoking and alcohol consumption) was significantly associated with the risk of development of both diseases. Such a relationship was not present for right 2D:4D and Dr–l (Tables 6 and 7).

**Table 6:** Results of logistic regression examining the relationships between 2D:4DR, 2D:4DL, Dr–l and leukoplakia

Sex	Model	Predicators^1^	Leukoplakia vs. control group				
			*β*	Standard error	Wald test	*p*	Nagelkerke’s *R*^2^
Men	1	2D:4D R	1.89	5.92	0.10	0.7487	0.3384
	2	2D:4D L	−7.11	6.48	1.20	0.2730	0.3433
	3	Dr–l	10.76	7.10	2.30	0.1297	0.3486
Women	1	2D:4D R	−5.52	6.63	0.69	0.4051	0.4119
	2	2D:4D L	−19.05	8.70	4.80	< 0.05	0.4417
	3	Dr–l	8.29	7.08	1.37	0.2417	0.4164

^1^adjusted for smoking and drinking alcohol

*β* logistic regression coefficient, *Nagelkerke's R*^*2*^ the value of logistic regression model

**Table 7:** Results of logistic regression examining the relationships between 2D:4DR. 2D:4DL, Dr–l and laryngeal cancer

Sex	Model	Predicators^1^	Laryngeal cancer vs. control group				
			*β*	Standard error	Wald test	*p*	Nagelkerke's *R*^2^
Men	1	2D:4D R	−2.15	6.89	0.10	0.7550	0.3657
	2	2D:4D L	−10.79	7.45	2.09	0.1479	0.3779
	3	Dr–l	10.43	8.30	1.58	0.2092	0.3748
Women	1	2D:4D R	−7.27	6.16	1.39	0.2378	0.3178
	2	2D:4D L	−20.55	8.02	6.56	< 0.05	0.3534
	3	Dr–l	9.77	7.53	1.68	0.1948	0.3195

^1^adjusted for smoking and drinking alcohol

*β* logistic regression coefficient, *Nagelkerke's R*^*2*^ the value of logistic regression model

**Fig. 1:**
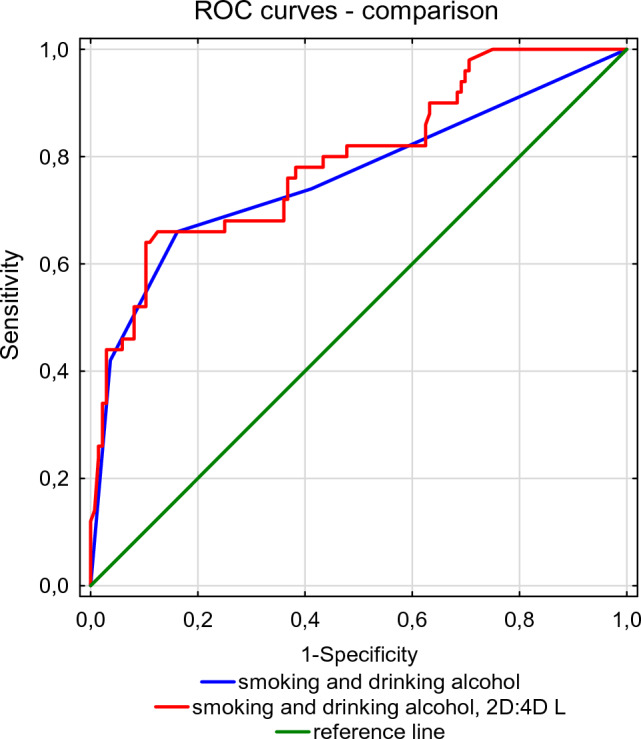
Comparison of ROC curves for models estimating the probability of leukoplakia in women

**Fig. 2:**
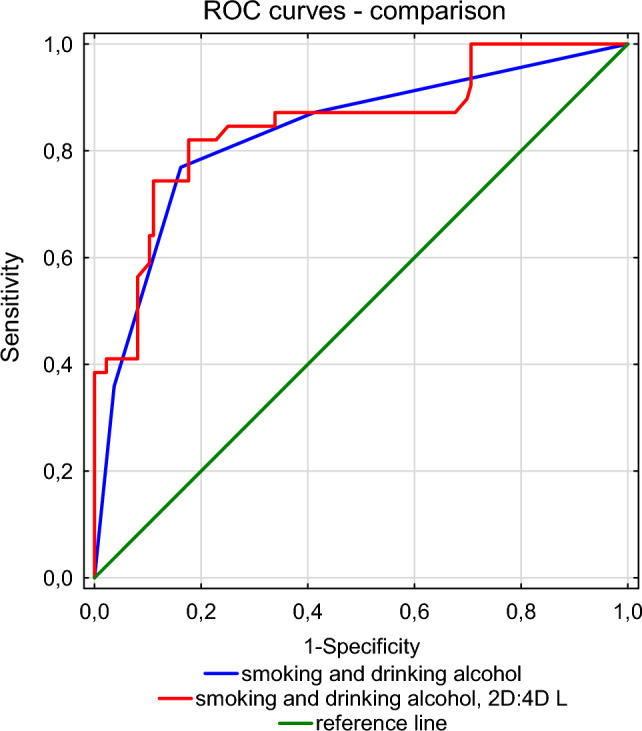
Comparison of ROC curves for models estimating the probability of laryngeal cancer in women

Predictive models estimating the risk of leukoplakia and laryngeal cancer in women, based solely on predictors such as smoking and alcohol consumption had a lower area under the ROC curve (AUC) than the model with the additional variable–left 2D:4D. After adding left 2D:4D, AUC for the leukoplakia model estimating the likelihood of leukoplakia increased from 0.8328 to 0.85, and in the case of laryngeal cancer from 0.762 to 0.7926 (Table 8). Even though in both cases the increase in AUC was not statistically significant, it is worth noting that in case of laryngeal cancer it was close to the threshold of significance, which suggests that left 2D:4D may prove to be a useful predictor of the development of this disease in women (Figs. 1 and 2).

**Table 8:** Comparison of predictive models for leukoplakia and laryngeal cancer in women

Predicators in model logistic regression	Leukoplakia vs. control group				Laryngeal cancer vs. control group			
	AUC	SD AUC	*Z*	*p*	AUC	SD AUC	*Z*	*p*
smoking (yes vs. no) drinking alc. (yes vs. no)	0.833	0.039	1.44	0.15	0.762	0.043	1.76	0.079
smoking (yes vs. no) drinking alc. (yes vs. no) 2D:4DL	0.85	0.039			0.793	0.039		

*AUC* Area under the ROC curve, *SD* standard deviation, *Z* test Z, *p* probability

## Discussion

Squamous cell carcinomas (SCC) of the larynx are the most common malignancies of the upper aerodigestive tract in the Western hemisphere (Oukessou et al. 2022). Laryngeal cancer constitutes 30% to 40% of all malignant head and neck tumors and 1% to 2.5% of all malignant neoplasms in the human body (Markou et al. 2013). Reported malignant transformation rates of laryngeal dysplasia vary between 11 and 25%; however, identifying which patients would transform into carcinoma is complex (Chen et al. 2019). The association between laryngeal leukoplakia, the most prevalent premalignant laryngeal lesions, and cancer has been the subject of numerous clinical studies and meta-analyses in recent decades (Kostev et al. 2018). The malignancy potential of the laryngeal lesions is also one of the major concerns of surgeons about choosing the treatment options, forming surgical margins, and deciding the follow-up periods. Finding biomarkers to overcome these concerns is an ongoing challenge (Tuncturk et al. 2021).

It is difficult to assess the validity of individual etiological factors, however, it seems that interaction of various risk factors has the largest contribution to cancer development. Environmental, exogenous and endogenous factors, as well as individual factors, including genetic predisposition, contribute to the development of cancer (Schulz et al. 2008). Tobacco smoking and alcohol consumption (irrespective of the type of beverage) have been established as the main etiologic risk factors for LC (Licitra et al. 2003). Tobacco dominates the risk for cancers of the vocal cords and glottis, alcohol is more prominent for cancer in the supraglottic region (Glade 1999). The study by Garavello et al. (2012) analyzed the effect of family history of laryngeal cancer and demonstrated that it was stronger in smokers and heavy drinkers (Garavello et al. 2012). Among other risk factors reported in literature are asbestos exposure, industrial pollution, and inadequate intake of anti-oxidant micronutrients found in fresh fruit and vegetables (Sheahan 2014). A meta-analysis conducted by Li et al. suggested a significantly increased risk of SCC associated with HPV infection and HPV-16 was the most frequently observed subtype in laryngeal cancer specimens and showed a strong association with the development of cancer (Li et al. 2013). In the last three decades, evidence has been provided by a number of researchers that sex hormone receptors are expressed in laryngeal carcinomas (Bianchini et al. 2008) and the accumulated research evidence has confirmed that laryngeal cancer is a hormone responsive cancer, comparable to other more researched secondary sex hormone cancers (Verma et al. 2020).

To the best of our knowledge, the only study that tried to investigate the hypothesis of genetic predisposition of LC using anthropometric measurements was conducted in 2005 (Rudić et al. 2005). The authors studied the relation between laryngeal cancer and quantitative digito-palmar dermatoglyphs features. The embryological development of the larynx starts at about the same time and from the same embryological layers as the development of the skin, from which the dermatoglyphs develop, therefore, changes in embryological period could have influences on development of the cancer but they could also have reflection on the expression of the dermatoglyphic patterns. The study comprised 40 male patients with the confirmed diagnosis of squamous cell cancer of the larynx, and a control group of 100 phenotypically healthy subjects. The study did not reveal any significant difference between case and control groups (Mardanshahi et al. 2017; Rudić et al. 2005).

To date, the relationship of 2D:4D ratio and laryngeal cancer has not been considered. Digit ratio studies may add to the knowledge about the etiology and risk factors of sex steroid-dependent cancers. They may aid in the prediction of susceptibility of such diseases (Manning and Fink 2018). A meta-analysis by Bunevicius (2018) found that prenatal sex hormones may influence the risk of developing prostate cancer and brain tumors (low ratio) and breast cancer as well as younger age of presentation of breast cancer, cervical dysplasia and brain tumors (higher ratios) (Bunevicius 2018; Bunevicius et al. 2016; Hong et al. 2014; Muller et al. 2011). Moreover, gastric cancer risk in Chinese women appeared to be correlated with low 2D:4D. This was consistent with the observation that estrogen may inhibit the development of gastric cancer (Freedman et al. 2007; Wang et al. 2018). Similarly, in our study concerning 2D:4D in patients with lung cancer, we hypothesized that prenatal estrogens may be protective against lung cancer in later life. We found that women with lung cancer have lower 2D:4D and that women with lower 2D:4D present earlier with the cancer than those with higher 2D:4D. Our data suggested that masculinized right 2D:4D may indicate a predisposition to lung cancer and to the more aggressive forms of lung cancer, both in women and men (Kasielska-Trojan et al. 2020). A recent study by (Ying et al. 2022) reported that 2D:4D lower than 1 was associated with higher oral cancer risk after accounting for several confounders. The authors concluded that intra-utero hormonal levels are associated with oral cancer risk. Positive results of the aforementioned studies provided a rationale for the current study in that we considered 2D:4D in women and men with laryngeal cancer and precancerous lesions—leukoplakia. This is the first study in the literature to include patients with both precancerous laryngeal lesions and laryngeal cancer. We found that left 2D:4D may prove to be a useful predictor of the development of these diseases in women. There are some controversies concerning the differences in the ratio sides. This may be related to a natural asymmetry in hormone receptor concentration. Some authors have claimed that right-hand 2D:4D is a better marker than left-side 2D:4D (Rahman et al. 2011; Hönekopp and Watson 2010), while others found both important. Moreover, Manning and Leinster (2001) reported only left 2D:4D as an indicator of age-dependent risk for female breast cancer. Here, we also found that left-side 2D:4D may be better indicator of the risk of laryngeal cancer in women. The differences between males and females continue throughout prenatal development to adulthood. These differences generate a lot of contrasts, and include, among others, variation in susceptibility for some diseases (Tevfik Dorak and Karpuzoglu 2012). Laryngeal cancer is among the neoplasms with the greatest gender differences found in most populations worldwide (Oukessou et al. 2022) and the site of LC differs widely according to gender as well. Women are more likely to have cancer of the supraglottis than of the glottis while in men the cancer is more frequently observed in the glottis (Bradford et al. 2020). Given the significant differences in how laryngeal cancer affects the two genders, it has been speculated that the difference results from the different susceptibilities of the tumor cells to steroid sex hormones (Hagedorn and Nerlich 2002). Oukessou et al. (2022) point out that the proportions of women in published studies on the subject of LC are limited, and it is necessary to seek for etiopathogenic factors involved in laryngeal cancer in women, especially in those without any significant risk factors (smoking and alcohol consumption).

Although the well-known and verified risk factors (smoking and alcohol consumption; HPV infection) proved to be the most important predictors of the diseases in our study, 2D:4D should also be taken into consideration while estimating the overall risk of laryngeal cancer. Moreover, our results may add to the knowledge on role of sex hormones in the carcinogenesis mechanisms in case of epithelial cancers. This may be related to different susceptibility to epithelial cancers in response to carcinogens as with smoking. Smoking can also have an effect on circulating testosterone levels. It was reported that smoking causes an acute increase in testosterone. This may result from the influence of cotinine, a tobacco metabolite, which inhibits testosterone breakdown (Zhao et al. 2016). In the light of such interpretation markers of sex hormone exposure may be important in estimating the risk of epithelial cancers of lungs, larynx and oropharynx.

Limitations: The presented study has some limitations. First, we included a limited number of patients (mainly with laryngeal cancer). Although the power of the performed analysis is good and the sample is sufficient for the analysis, to verify our preliminary observation studies on a larger number of participants are needed for external validation of the models. Also, inclusion of patients of different ethnicity would be valuable, as 2D:4D shows ethnic differences, in this regard it is similar to some other risk factors influencing LC occurrence. Due to the small number of participants, we did not analyze interactions of stimulants use and left 2D:4D in the context of the impact on the risk of developing laryngeal cancer and leukoplakia.

## Conclusions

1. Low left 2D:4D is associated with an increased risk of developing leukoplakia and laryngeal cancer in women.
2. Correlations between left 2D:4D and leukoplakia seem to be more important in statistical rather than clinical aspects (weak correlations). However, in the case of laryngeal cancer, left 2D:4D may serve as additional variable (to other known risk factors, such as smoking and/or alcohol consumption) which can improve cancer risk prediction.
3. The results presented in this study point to the necessity to further investigate the relationships between LC and premalignant laryngeal lesions and genetic and hormonal factors that could be represented by 2D:4D.

## Data Availability

Available on request from the corresponding author.
